# Carbapenem Resistance Mediated by Oxacillinases and Metallo-β-Lactamases in Acinetobacter baumannii Isolated From Patients in Critical Care Units

**DOI:** 10.7759/cureus.110123

**Published:** 2026-06-02

**Authors:** MuthuLakshmi BackiaSubramanian, Madhumala Shanmugasundaram, Shanthi Mariappan, Uma Sekar, Renuka MK, Thyagarajan P Ravinder, Rhea Michelle J Khodabux, Sribal Selvarajan

**Affiliations:** 1 Department of Microbiology, Sri Ramachandra Institute of Higher Education and Research, Chennai, IND; 2 Department of Critical Care Medicine, Sri Ramachandra Institute of Higher Education and Research, Chennai, IND; 3 Department of Microbiology, Kilpauk Medical College, Chennai, IND; 4 Department of Microbiology, Vels Medical College and Hospital, Chennai, IND

**Keywords:** acinetobacter baumannii, carbapenem resistance, critically ill patients, healthcare, multidrug resistance

## Abstract

Introduction

*Acinetobacter baumannii* (*A. baumannii*) is a multidrug-resistant (MDR) pathogen that causes nosocomial infections, especially in ICU settings. Carbapenem resistance among *A. baumannii* occurs due to the production of metallo-β-lactamases (MBLs) and OXA-carbapenemases. This study was undertaken to detect the presence of OXA-carbapenemase and MBL by phenotypic and genotypic methods.

Materials and methods

This in vitro, laboratory-based prospective study was conducted on 190 isolates collected from the clinical microbiology division of a 1,600-bed university teaching hospital from August 2023 to August 2024.

Results

A total of 190 isolates were obtained from the ICUs. The majority of these were isolated from respiratory secretions (77). Carbapenem resistance was detected in 161 isolates. All the carbapenem-resistant isolates exhibited positive Modified Hodge test (MHT) results, while 147 isolates were MBL screen test positive. Among the *bla*OXA genes, the *bla*_OXA-23-like_ gene was predominantly detected (43); in combination with *bla*_OXA-51-like_, it was detected in 77 isolates. The *bla*_OXA-24-like_ gene was not detected in this study. Among the MBL genes, *bla*_NDM_ was detected in the majority of isolates (12). Fourteen isolates did not harbor any of the genes looked for in the study.

Conclusion

The increase in carbapenem resistance in *A. baumannii* infections limits the clinical efficacy of antibiotics used in treatment. Therefore, these infections are a significant threat in hospital settings.

## Introduction

*Acinetobacter baumannii *is a gram-negative pathogen that is opportunistic in nature. It can survive and flourish in hospital environments and thrive in harsh conditions [1]. Hence, *A. baumannii* proves to be a notable pathogen in healthcare settings, responsible for causing various infections like pneumonia, bacteremia, UTIs, and wound infections [2]. The two main therapeutically significant traits of *A. baumannii* are its high level of natural resistance to antibiotics and its exceptional capacity to acquire both pertinent foreign resistance mechanisms and upregulate its innate antimicrobial resistance mechanisms [3]. This organism is associated with an alarmingly high mortality rate. This increased mortality rate is attributed to a plethora of reasons, among which resistance to carbapenems plays a major role [4]. Carbapenems are crucial antimicrobial agents in the treatment of nosocomial infections because of their high potency against gram-negative organisms [5]. Carbapenem-resistant *A. baumannii* (CRAB) develops resistance by several collaborative mechanisms, such as hydrolysis by beta-lactamases, modifications to outer membrane proteins and penicillin-binding proteins, and enhanced activities of efflux pumps [6]. CRAB isolates are known for their production of an assortment of carbapenemase enzymes on mobile genetic elements that can spread through horizontal gene transfer. The most common carbapenemase enzymes include Ambler class D or OXA-carbapenemases and Ambler class B or metallo-β-lactamases (MBLs) [7]. There are four main families of OXA genes involved in resistance against carbapenems, which include *bla*_OXA-23-like_, *bla*_OXA-24-like_, *bla*_OXA-51-like_, and *bla*_OXA-58-like_ genes. Among the MBL genes, *bla*_NDM_, *bla*_IMP_, and *bla*_VIM_ are the most common.

Most studies on *A. baumannii* have focused on phenotypic resistance patterns or individual resistance genes. There is a paucity of regional data from tertiary care settings on the molecular detection and co-occurrence of both OXA-type carbapenemases and MBLs, such as *bla*_NDM_, *bla*_IMP_, and *bla*_VIM_, in *A. baumannii* clinical isolates. Moreover, limited studies have systematically examined the combined presence of these genes within a single isolate, which has implications for treatment failure and infection control. This study aims to address this gap by detecting and characterizing the presence and co-existence of these carbapenem resistance genes in *A. baumannii* isolated from clinical specimens.

## Materials and methods

Bacterial strains

Study Design

This was an in vitro, laboratory-based prospective study.

Study Setting

The study was conducted in a 1,600-bed university teaching hospital from August 2023 to August 2024.

Sample Size

It included 190 non-duplicate, clinically significant *A. baumannii* isolates recovered from clinical specimens of patients hospitalized in ICUs, of which 161 isolates were carbapenem-resistant.

Specimen Types

The isolates were obtained from various clinical samples, such as lower respiratory tract secretions, including endotracheal secretions, bronchoalveolar lavage, and bronchial wash; wound exudates; pus; blood; and urine.

Criteria for Clinical Significance

The patient’s clinical history, Gram stain showing the presence of the organism, presence of intracellular forms of the organism, and growth in pure culture with a notable colony count were taken into consideration to determine the clinical significance of the isolates.

Ethics Approval and Date

The study was approved by the Institutional Ethics Committee (IEC-NI/23/AUG/88/42) on 25/08/2023.

Antimicrobial susceptibility testing

Disk Diffusion Method

Following the recommendations set forth by the Clinical and Laboratory Standards Institute (CLSI), the disk diffusion method was used to evaluate susceptibility to various classes of antibiotics. The antibiotics tested were amikacin (30 µg), ciprofloxacin (5 µg), ceftazidime (30 µg), piperacillin-tazobactam (100/10 µg), imipenem (10 µg), and meropenem (10 µg) from HiMedia Laboratories (Mumbai, Maharashtra, India). Susceptibility to tigecycline was tested using tigecycline disks (15 µg) from Tulip Diagnostics (Goa, India). The tigecycline susceptibility breakpoint criteria established by the US FDA were used to evaluate zone diameters. Susceptibility to colistin was tested using the microbroth dilution method (Tulip Diagnostics, Goa, India). A minimum inhibitory concentration (MIC) of ≤2 µg/mL was considered intermediate, and an MIC of ≥4 µg/mL was considered resistant according to CLSI guidelines.

Detection of Carbapenemases

The Modified Hodge test (MHT) was performed to detect the production of carbapenemases. A fresh 24-hour overnight culture of *Escherichia coli* (*E. coli*) ATCC 25922 was inoculated in peptone water and incubated for 15 minutes. This inoculum was compared with the 0.5 McFarland standard and adjusted according to the turbidity. A lawn culture was made on Mueller-Hinton agar, and imipenem disks (10 µg) were placed at the center of the test area. An overnight culture of the test organism was streaked in a straight line from the edge of the disk to the edge of the plate. This setup was incubated at 37°C for 24 hours. The presence of distorted zones was interpreted as a positive result. MHT is not recommended by CLSI as a standard technique for the detection of carbapenemase production; however, several authors have found this test useful and affordable for use [6].

Detection of Metallo-Beta-Lactamases

The detection of metallo-beta-lactamase production was done by the ethylenediaminetetraacetic acid (EDTA) impregnation method. A 24-hour overnight culture of the test organism was lawn cultured on Mueller-Hinton agar. Two imipenem 10 µg disks were placed at an equal distance. A 0.5 M EDTA solution was prepared by dissolving 186.1 g of disodium EDTA in 1,000 mL of distilled water, and the pH was maintained at 7. One of the imipenem disks was impregnated with 5 µL of EDTA solution and incubated at 37°C for 24 hours. If the zone diameter difference between the impregnated disk and the normal disk was greater than 7 mm, the results were interpreted as positive [8].

Molecular detection of OXA and MBL genes using PCR

DNA Extraction

The DNA of the collected isolates was extracted using the boiling lysis procedure. The individual colonies were suspended in 400 µL of TE buffer or distilled water. This suspension was boiled at 95°C for 10 minutes and immediately frozen at -20°C for another 10 minutes. This was then centrifuged at 12,000 rpm for 10 minutes. The suspension was collected, and the pellet was discarded [9]. A 2 µL aliquot of this suspension was used as the template for amplification. The suspension was stored at -20°C for further analysis.

Multiplex PCR for OXA-Carbapenemase Genes

Multiplex PCR was performed for all the isolates for the detection of *bla*_OXA-23-like_ genes (*bla*_OXA-23_, *bla*_OXA-27_, and *bla*_OXA-49_), *bla*_OXA-24-like_ genes (*bla*_OXA-24_, *bla*_OXA-25_, *bla*_OXA-26_, and *bla*_OXA-40_), *bla*_OXA-51_, and *bla*_OXA-58_. The primers, amplification conditions, and product size of each gene were obtained from the reference study [10].

PCR for Metallo-Beta-Lactamase Genes

All the isolates were subjected to PCR for the detection of MBL genes, namely *bla*NDM, *bla*VIM, and *bla*IMP. The primers, amplification conditions, and product size of each gene were adopted from previously published studies [11,12].

After amplification, visualization of the bands was carried out using 2% agarose gel electrophoresis with ethidium bromide (EtBr). Strains previously confirmed to carry the specific gene using PCR and gene sequencing were used as positive controls, and *E. coli* ATCC 25922 was used as the negative control for optimization.

DNA Sequencing

Representative isolates were subjected to automated DNA sequencing. Analysis of the DNA sequences was done using BioEdit software. Similarity checks were performed using Nucleotide BLAST (BLASTN), and the sequences were submitted to GenBank, after which accession numbers were obtained.

Data presentation and software

Data were analyzed using Microsoft Excel (Microsoft Corp., USA). Graphs were generated using Microsoft Word (Microsoft Corp., USA).

## Results

Specimen distribution

The 190 isolates of *A. baumannii *were distributed among various clinical specimens, namely respiratory secretions (n = 77), exudative specimens (n = 50), blood (n = 50), and urine (n = 13). Of these, 161 isolates were resistant to carbapenem. The isolates were predominantly obtained from males (n = 140, 73.7%) compared to females (n = 50, 26.3%).

Antimicrobial susceptibility test

Antimicrobial susceptibility testing was performed for all 190 isolates. The resistance patterns for various classes of antibiotics were noted as follows: ceftazidime, 87.3% (n = 166); ciprofloxacin, 86.8% (n = 165); piperacillin-tazobactam, 85.8% (n = 163); amikacin, 84.2% (n = 160); imipenem, 84.7% (n = 161); and meropenem, 84.7% (n = 161).

**Figure 1:**
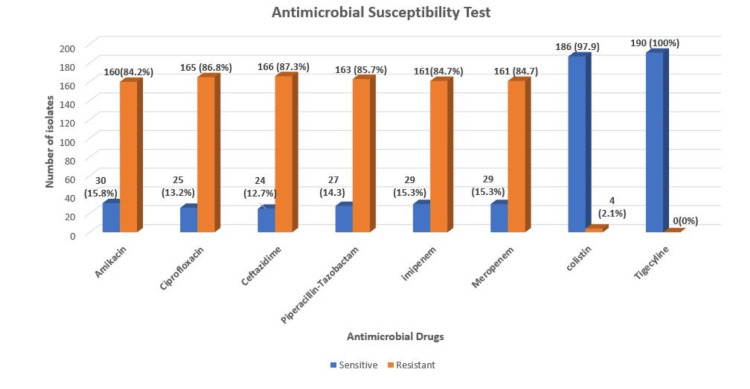
Antimicrobial susceptibility patterns of Acinetobacter baumannii isolates.

All the isolates were found to be susceptible to tigecycline, while 2.1% (n = 4) were found to be resistant to colistin by the broth microdilution method (Figure 1).

**Figure 2:**
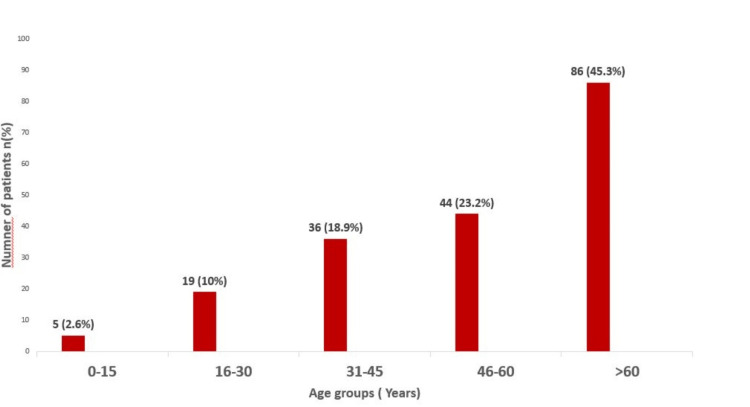
Age-wise distribution of patients.

The age distribution of both male and female patients is shown in Figure 2. The median age of the study population was found to be 58 years.

ICU stay was calculated as the duration from ICU admission to discharge or death. The median ICU stay among patients with CRAB infection was 12 days (IQR: 7-22). The largest proportion of patients were admitted under general medicine (21.1%), followed by critical care medicine (14.9%), neurology (14.28%), and respiratory medicine (10.5%).

The diagnosis-wise distribution is shown in Table 1. The highest number of cases presented with sepsis/septic shock (49%), followed by pneumonia and other respiratory ailments (16.7%).

**Table 1:** Diagnosis-wise distribution of Acinetobacter baumannii isolates. CRAB: Carbapenem-resistant *Acinetobacter baumannii*; AKI: Acute kidney injury.

Final diagnosis	No. of CRAB isolates (n = 161)	Percentage (%)
Sepsis/septic shock	79	49
Pneumonia/pneumothorax/respiratory failure/alveolar hemorrhage	27	16.8
Traumatic brain injury	12	7.5
Skin and soft tissue infections (cellulitis, abscess, diabetic foot ulcer, necrotizing fasciitis)	11	6.8
Urinary tract infection/AKI/CKD	8	5
GI bleed/GI perforation/GI ulcer	8	5
Burn wound infection	5	3.1
Malignancy	5	3.1
Bacterial meningitis/encephalitis	5	3.1
Immunosuppressed state due to transplantation	1	0.6
Total	161	100

Prior antibiotic exposure to agents such as cephalosporins, beta-lactam/beta-lactamase inhibitors, aminoglycosides, and fluoroquinolones was observed in 144 (89.4%) patients before the isolation of CRAB strains. Of these, 56 (34.7%) had received carbapenems.

Polymerase chain reaction

Among the 161 CRAB isolates, which were also MHT-positive, 134 (83.2%) carried one or more OXA carbapenemase-encoding genes. The genes identified were *bla*_OXA-23-like_, *bla*_OXA-51-like_, and *bla*_OXA-58-like_. Among these, the *bla*_OXA-23-like_ gene was predominantly present (n = 43), followed by *bla*_OXA-51-like_ (n = 14) and *bla*_OXA-58-like_ (n = 1). Coexistence of multiple OXA genes was noted in 76 isolates. The *bla*_OXA-24-like_ gene was not detected in any of the study isolates.

Distribution of metallo-beta-lactamase-encoding genes

Two MBL genes, *bla*_NDM_ and *bla*_IMP_, were identified in this study. Of the 190 isolates, 75 (38.9%) were positive for MBL genes: *bla*_NDM_ (n = 74) and *bla*_IMP_ (n = 1). Notably, 73 of the MBL-positive isolates belonged to the CRAB group: *bla*_NDM_ (n = 72) and *bla*_IMP_ (n = 1).

**Figure 3:**
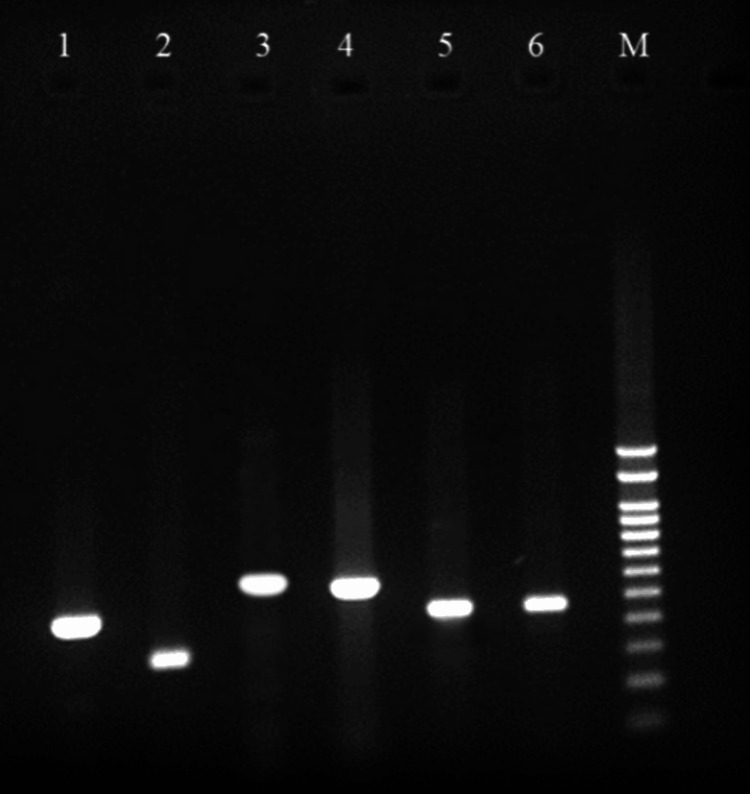
Representative agarose gel electrophoresis image showing OXA and MBL gene amplicons. Lane 1: *bla*_OXA-23-like_, 501 bp Lane 2: *bla*_OXA-51-like_, 353 bp Lane 3: *bla*_OXA-58-like_, 599 bp Lane 4: *bla*_IMP_, 587 bp Lanes 5 and 6: *bla*_NDM_, 495 bp M: Marker/ladder.

Among the 161 CRAB isolates, 134 harbored OXA genes, while among the remaining 27 OXA-negative isolates, 12 carried the NDM gene and one carried the IMP gene. The *bla*_VIM_ gene was not detected in any of the study isolates. Fourteen isolates did not carry any of the OXA or MBL genes explored in this study. Coexistence of MBL and OXA genes was seen in 27 isolates. The results were visualized using agarose gel electrophoresis, as shown in Figure 3. The detailed breakdown of the distribution of OXA and MBL genes is listed in Table 2.

**Table 2:** Distribution of carbapenemase gene patterns (OXA and MBL) among the study isolates.

Gene pattern	No. of isolates (%)
OXA alone	107 (66.5)
NDM alone	12 (7.5)
IMP alone	1 (0.6)
NDM and OXA	27 (16.7)
None	14 (8.7)
Total	161 (100.0)

The sequences were submitted to GenBank, and the following accession numbers were obtained: *bla*_OXA-23_ (PQ55937), *bla*_OXA-51_ (PQ559636), *bla*_OXA-58_ (PQ568953), *bla*_NDM_ (PV931735), and *bla*_IMP_ (PX353796).

Phenotypic tests

**Figure 4:**
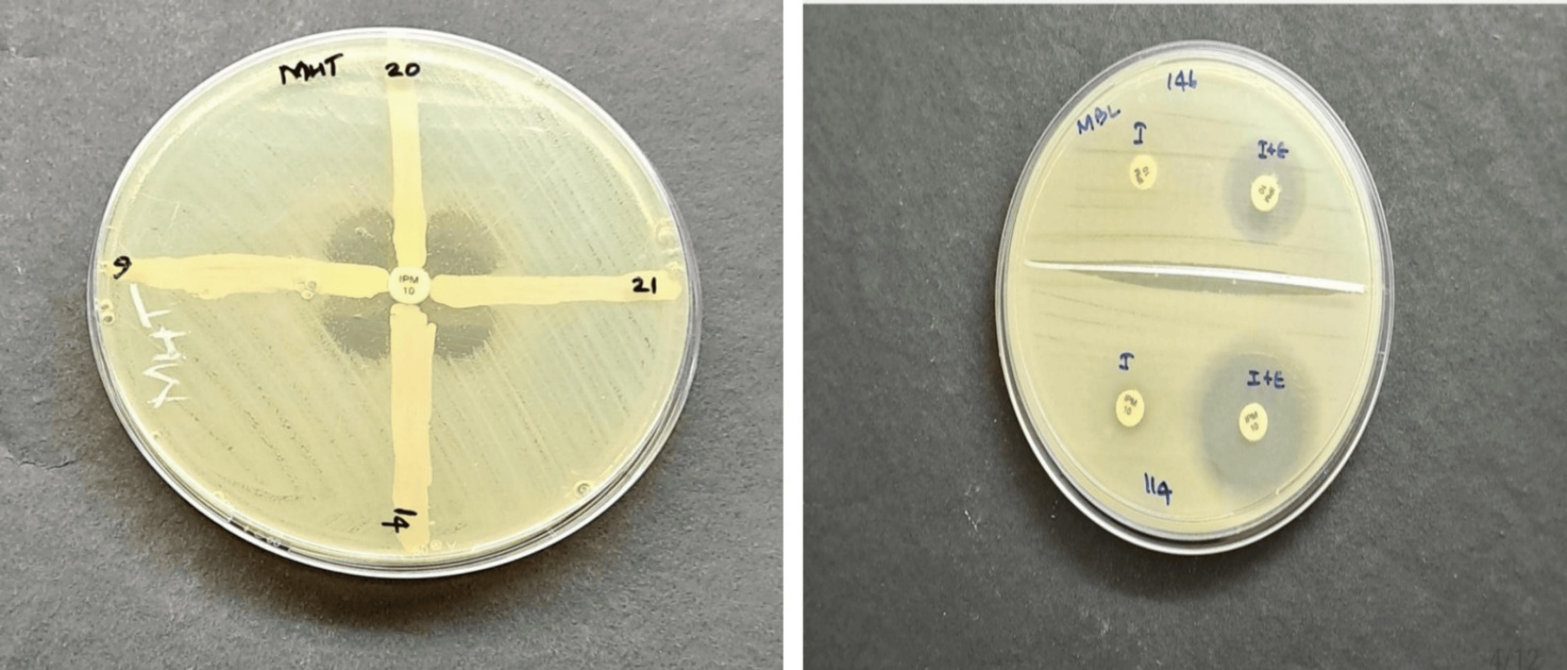
Representative image of phenotypic carbapenemase detection tests: Modified Hodge test and metallo-beta-lactamase test.

The Modified Hodge test exhibited positive results for 161 (84.2%) isolates among the 190 isolates tested. All 161 isolates were resistant to imipenem. MBL screening with EDTA was positive for 77.3% (n = 147/190) of isolates (Figure 4).

**Figure 5:**
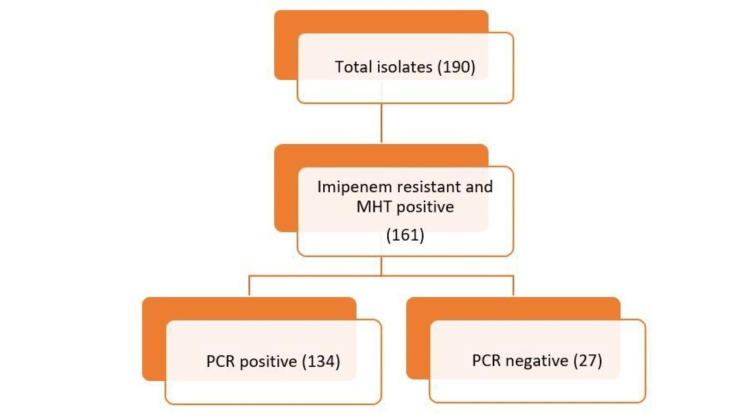
Flowchart showing the distribution of resistance genes among Modified Hodge test (MHT)-positive isolates.

 The distribution of genes in MHT- and MBL-positive isolates is shown in Table 3 and Figure 5.

**Table 3:** Distribution of resistance genes among metallo-beta-lactamase (MBL)-positive isolates.

	PCR-positive (OXA/NDM/IMP genes detected)	PCR-negative (OXA/NDM/IMP genes not detected)	Total
MBL-positive	73	74	147
MBL-negative	2	12	14
Total	75	86	161

## Discussion

*Acinetobacter baumannii* has been recognized as one of the most significant organisms causing nosocomial infections in patients with prolonged hospital stays. Since the identification of the carbapenem class of antibiotics in the 1980s, carbapenems have been considered a major treatment option for severe infections caused by *A. baumannii*, and the rising incidence of CRAB isolates has been regarded as a great public health concern in recent years. Carbapenem resistance occurs mainly due to two mechanisms, namely the production of class D oxacillinase enzymes and class B beta-lactamases.

Sample distribution

In this study, 190 isolates were collected from patients admitted to ICUs. Of these, 161 were CRAB isolates, most of which were collected from critically ill patients, making the organism one of the most important healthcare-associated pathogens. This finding was similar to a study done in Morocco [13]. In agreement with other studies, this organism was mostly recovered from respiratory secretions (n = 77). The respiratory secretions consisted of endotracheal secretions (58), tracheal aspirate (8), pleural fluid (4), sputum (4), and bronchial wash (3). The isolation rate of *A. baumannii* is high in respiratory samples because it is known to mainly cause infections in the lower respiratory tract [14].

Gender, age, and diagnosis-wise distribution

The organism was more frequently isolated from male patients than from female patients. This finding is in accordance with previous Indian studies [15,16]. However, a 2025 study from western India reported a higher number of CRAB isolates from female patients [17]. This study also showed notable predominance among elderly patients (age >60 years) requiring prolonged critical care [15].

Supporting the findings of earlier studies, we noted the average length of ICU stay in this study to be 12 days [15,18]. Antimicrobial resistance was found to be associated with numerous factors, including male gender, age above 50 years, prolonged ICU stay, prior antibiotic use, and underlying respiratory ailments.

Several studies from Indian ICUs have documented a high burden of sepsis. Cross-sectional studies from 35 ICUs showed a high point prevalence of sepsis and substantial antimicrobial resistance, which was consistent with the findings of this study [19]. Another large multicenter prospective registry of over 1,000 patients reported a significant septic shock burden of over 30% [15]. Sepsis patients additionally exhibited higher rates of respiratory tract infections [18].

A substantially high proportion of carbapenem resistance, 161 (84.7%), was observed among the study isolates, comparable to previously published studies from India [6,20]. Studies from South Korea and Morocco reported 87.4% and 94% carbapenem resistance, respectively [13], and another study from Thailand reported 100% resistance to imipenem and 98.91% resistance to meropenem [21].

Tigecycline demonstrated 100% susceptibility across all isolates. The majority of the strains were susceptible to colistin; however, a small proportion of strains were resistant (2.1%). This was in accordance with colistin resistance data from European studies (0%-4%) [22]. A rise in resistance to colistin and tigecycline is becoming a significant concern.

In this study, OXA carbapenemase genes were encountered in 134 isolates. Out of the four OXA families, we report *bla*_OXA-23_ as the most common gene (26.7%) responsible for carbapenem resistance. This finding was in agreement with reports from India [23,20,6,24,17,16], Pakistan [25], and Thailand [21]. Various studies from countries such as China, Morocco, and Iran have also noted that *bla*_OXA-23_ is the most common carbapenem resistance-mediating gene [26,27].

The *bla*_OXA-51-like_ gene cluster is encoded chromosomally and is highly prevalent. In this study, it was the second most common OXA carbapenemase gene (8.6%). Studies have also reported co-occurrence of *bla*_OXA-23_ and *bla*_OXA-51_ (45.96%) [6]. The *bla*_OXA-58-like_ gene (1.7%) was the least detected in this study, being present only in three isolates. It coexisted with other OXA genes. None of the isolates harbored the *bla*_OXA-24_ gene. This result was consistent with a study from India [17]. bla_OXA-24_ and blaOXA-58 have been reported from other countries, but not many Indian reports provide information on the prevalence and distribution of these genes. A study from Mangalore, South India, reported seven strains possessing the *bla*_OXA-58-like_ gene cluster [23]. Another study by Amudhan SM et al. [6] reported detection of *bla*_OXA-24-like_ in two isolates and *bla*_OXA-58_ in one isolate. Sharma M et al. [24] and Gupta P et al. [16], in their studies in 2021 and 2025, reported the presence of the *bla*_OXA-58_ gene in 2% and 4.76% of their isolates, respectively. In contrast to the above reports, Karunasagar A et al. [23] from India reported a very high proportion of strains possessing the *bla*_OXA-24-like_ gene (22.9%) and *bla*_OXA-58_ (4.2%).

Among the class B metallo-β-lactamase genes, *bla*_NDM_ and *bla*_IMP_ were detected in this study. Within the CRAB isolates that did not show any OXA genes, MBL genes were detected in 13 isolates, of which the *bla*_NDM_ gene was predominantly identified in 12 isolates. One isolate harbored the *bla*_IMP_ gene. The *bla*_NDM_ gene was first isolated in 2009 in New Delhi, India. Since then, India has been the epicenter of this gene. Various studies from India have reported similar results [28,29,20]. Some authors have reported *bla*_VIM_ to be the most common MBL gene [27,24,15]; however, in this study, *bla*_VIM_ was not detected. *bla*_IMP_ was found in only one isolate. Similar to this study, a report from West Bengal documented the absence of *bla*_VIM_ [29].

Coexistence of MBL and OXA genes was detected in 27 isolates. All 27 isolates were found to have one or more OXA genes with *bla*_NDM_. *bla*_IMP_ in this study was found only in one isolate, which did not harbor any of the *bla*_OXA_ genes or *bla*_NDM_. Studies have reported similar findings from Iran, India, and Pakistan [27,12,25,26].

Comparison between the phenotypic (MHT) and genotypic (PCR) methods revealed that, among 161 CRAB isolates, 134 showed phenotypic positivity by MHT, whereas 27 isolates were phenotypically negative. Of the 27 PCR-negative isolates, 13 carried MBL genes (*bla*_NDM_ = 12, *bla*_IMP_ = 1). The remaining 14 isolates did not possess any of the genes detected in this study. This may be due to the presence of other OXA or MBL genes, such as SIM, GIM, and SPM [30].

Similarly, on comparing the MBL test with PCR, among the 147 PCR-positive isolates, 73 were phenotypically positive and 74 were phenotypically negative. Two of the 14 PCR-negative imipenem-resistant isolates screened positive for MBL. This may be attributed to the presence of other MBL genes that were not looked for in this study, such as SPM, GIM, and SIM [30]. The remaining 14 isolates point to the presence of other mechanisms, such as efflux pumps or loss of porins in the cell wall, which also contribute to carbapenem resistance.

The use of carbapenems for treatment has declined in recent years because of the organism’s resistance profile against the antibiotic. Therefore, therapeutic options have been restricted to the antibiotic armamentarium of polymyxins and tigecycline. Surveillance should be done to limit the spread of CRAB from environmental sources and colonization of patient equipment. Infection control measures should be directed toward the source of infection; this will help curb the spread of the organism. Outbreaks of such MDR organisms can be controlled by proper hand hygiene, antibiotic stewardship, appropriate disinfection of patient care equipment, and other infection control policies.

Limitations

This study mainly focused on the most common carbapenemase resistance genes. Less prevalent genes, such as *bl*a_SIM_, *bla*_SPM_, and *bla*_GIM_, were not evaluated in this study. It was conducted in a single center with a limited sample size, which may affect generalizability. Other mechanisms contributing to carbapenem resistance, particularly efflux pump-mediated resistance and porin loss, were beyond the scope of the present study.

## Conclusions

The primary cause of carbapenem resistance in *A. baumannii* in this study is the acquisition of genes coding for OXA-type beta-lactamases. The contribution of OXA beta-lactamases to carbapenem resistance has been shown to have an important clinical impact on the treatment of nosocomial infections. The present investigation also identified the co-occurrence of two different types of carbapenem resistance genes. Strict infection control measures can help prevent the spread of CRAB, and early screening can help healthcare professionals better understand the mechanism of resistance and identify treatment options for patients.
